# Alveolar Soft Part Sarcoma in Pediatric and Young Adult Patients: A Report From the Children’s Oncology Group Study ARST0332

**DOI:** 10.1002/1545-5017.70228

**Published:** 2026-03-16

**Authors:** Jacquelyn N. Crane, Wei Xue, Amira Qumseya, Thomas Scharschmidt, Kathryn R. Tringale, Oscar Lopez Nunez, Jiaqi Hu, Donald A. Barkauskas, Rajkumar Venkatramani, Sheri L. Spunt, Aaron R. Weiss, Noah C. Federman

**Affiliations:** 1 Children’s Hospital of Philadelphia, Department of Pediatrics, Perelman School of Medicine, University of Pennsylvania, Philadelphia, Pennsylvania, USA; 2 Department of Biostatistics, College of Public Health and Health Professions and College of Medicine, Gainesville, Florida, USA; 3 Department of Orthopedic Surgery, Nationwide Children’s Hospital, Columbus, Ohio, USA; 4 Department of Radiation Medicine and Applied Sciences, University of California San Diego, La Jolla, California, USA; 5 Department of Pathology and Laboratory Medicine, University of Cincinnati College of Medicine, Cincinnati, Ohio, USA; 6 Department of Population and Public Health Sciences, Keck School of Medicine of the University of Southern California, Los Angeles, California, USA; 7 Division of Hematology/Oncology, Department of Pediatrics, Texas Children’s Cancer Center, Texas Children’s Hospital, Baylor College of Medicine, Houston, Texas, USA; 8 Department of Pediatrics, Stanford University School of Medicine, Stanford, California, USA; 9 Department of Pediatrics, MaineHealth Maine Medical Center, Portland, Maine, USA; 10 Department of Pediatrics, University of California Los Angeles David Geffen School of Medicine, Los Angeles, California, USA

**Keywords:** ASPS, NRSTS, sarcoma

## Abstract

**Background:**

Alveolar soft part sarcoma (ASPS) is a rare soft tissue sarcoma occurring most commonly in adolescence and young adulthood.

**Methods:**

We present the clinical characteristics, treatments, and outcomes of patients with newly diagnosed ASPS enrolled on the Children’s Oncology Group study ARST0332. Patients were treated with risk-adapted therapy, including surgery with or without radiotherapy and ifosfamide and doxorubicin.

**Results:**

Twenty-four patients with ASPS enrolled on ARST0332 between 2007 and 2012 were analyzed. The majority of primaries were extremity tumors (71%) and > 5 cm (54%). Nearly half (46%) of patients had metastatic disease at diagnosis, all of whom had primary tumors > 5 cm and pulmonary metastases with or without extrapulmonary metastases. Six patients were evaluable for response to neoadjuvant chemoradiotherapy without an objective response. Estimated 5-year event-free survival (EFS) and overall survival (OS) were 91% and 100% for low-risk (*n* = 11), 0% and 50% for intermediate-risk (*n* = 2), and 0% and 59% for high-risk disease (*n* = 11), respectively. EFS and OS differed significantly by maximal tumor diameter, presence or absence of metastatic disease, risk group, treatment arm, and upfront primary site resection status.

**Conclusions:**

Patients with low-risk ASPS (non-metastatic, grossly resected tumors ≤ 5 cm) had excellent outcomes with surgery with or without radiation on ARST0332. Chemotherapy has been reported to be generally ineffective in ASPS and, indeed, there were no objective responses to neoadjuvant chemoradiotherapy on ARST0332. All patients treated with combination chemoradiotherapy ultimately developed disease progression/relapse. A different therapeutic approach is needed for patients with unresectable or metastatic ASPS.

## 1 | Introduction

Alveolar soft part sarcoma (ASPS) is a rare soft tissue sarcoma that occurs most often in adolescents and young adults [1–4]. Most cases of ASPS are characterized by the unbalanced translocation der(17)t(X,17)(p11;q25) resulting in an *ASPSCR1::TFE3* fusion gene [5–7]. While ASPS generally has an indolent course, it is characterized by a high rate of metastatic spread with a propensity to metastasize to the lungs, bone, and central nervous system [1, 3, 8–10].

The standard management of localized ASPS includes complete surgical resection, when feasible [2, 11–13]. However, the optimal treatment approach to unresectable and/or metastatic ASPS has not been well defined. While radiation and conventional chemotherapy agents such as ifosfamide and doxorubicin are routinely used in neoadjuvant and adjuvant settings for many types of soft tissue sarcomas, data on radiation specifically in ASPS are limited and ifosfamide and doxorubicin have shown limited efficacy in the treatment of ASPS [3, 8–12, 14–26]. Data on chemo-responsiveness of ASPS and other types of non-rhabdomyosarcoma soft tissue sarcomas (NRSTS) in children was limited prior to the activation of ARST0332, a prospective clinical trial from the Children’s Oncology Group for children and young adults with NRSTS. Herein, we describe the clinical features, treatment, and outcomes of patients with ASPS enrolled on ARST0332.

## 2 | Methods

Patients less than 30 years of age with newly diagnosed NRSTS were enrolled on the Children’s Oncology Group (COG) study ARST0332. The eligibility criteria, treatment guidelines, and outcomes for all patients on ARST0332 have been previously reported [27]. This subset analysis included patients with ASPS enrolled on ARST0332. The study was approved by the institutional review boards at the participating institutions and enrolling patients and/or their legal guardian(s) provided written informed consent and/or assent before participation.

Central pathology review was performed for all cases enrolled in ARST0332. Tumors were graded using both the Pediatric Oncology Group (POG) and Fédération Nationale des Centres de Lutte Contre le Cancer (FNCLCC) systems. Although FNCLCC grading is not routinely applied to ASPS, it was uniformly assigned across all NRSTS to support standardized data collection and enable future histopathologic analyses. ASPS cases were classified as grade 3 per POG criteria, which was used for risk stratification and treatment assignment [28, 29].

For patients with ASPS, all defined as having high-grade disease, gross total resection of the primary tumor was required prior to study enrollment, unless resection posed unacceptable morbidity, the tumor was > 5 cm with expected gross or microscopic residual tumor post-resection, or metastatic disease was present. For this analysis, the extent of tumor resection was defined as R0 (complete resection), R1 (microscopic residual disease), or R2 (gross residual disease).

As shown in Figure S1, patients were classified into risk groups (low, intermediate or high) and treatment arms (Arms A, B, C, or D) based on the presence or absence of metastatic disease, extent of surgical resection, POG grade, and size of the primary tumor [27]. For patients with ASPS, the low-risk (LR) group included patients who had undergone R0 or R1 resection of a non-metastatic tumor ≤ 5 cm; the intermediate-risk (IR) group included patients who had undergone R0 or R1 resection of a non-metastatic tumor > 5 cm or had a unresected, non-metastatic tumor of any size; and the high-risk (HR) group included patients with metastatic disease. Patients with ASPS who had undergone R0 resection of a non-metastatic tumor ≤ 5 cm were assigned to treatment arm A with observation after surgery; those who had undergone R1 resection of a non-metastatic tumor ≤ 5 cm were are assigned to treatment Arm B with adjuvant radiotherapy (RT) after surgery; those who had undergone R0 or R1 resection of a non-metastatic tumor > 5 cm or R0 or R1 resection of a metastatic tumor were assigned to treatment arm C with adjuvant chemoradiotherapy; those who had undergone R2 or no resection of their primary site were assigned to treatment arm D with neoadjuvant chemoradiotherapy. As shown in Table S1, patients on arms C and D received ifosfamide and doxorubicin and primary site RT began at week 4. Patients on arm D had surgery during week 13 of protocol therapy, when feasible. When feasible, metastases were excised at the end of therapy and RT was given to all gross or microscopic residual metastases after maximal surgical resection.

Primary tumor response was assessed via volumetric (elliptical model) measurements, and metastatic response was assessed using Response Evaluation Criteria in Solid Tumors (RECIST) [30]. Overall imaging response at week 13 in patients on treatment arm D was coded as complete response (CR), partial response (PR), stable disease (SD), or progressive disease (PD). Patients in all treatment groups had posttreatment imaging surveillance over a 5-year period.

Patient and clinical characteristics, treatments, and events were summarized using descriptive statistics. Event-free survival (EFS) was defined as the time from study enrollment to disease progression or recurrence, second malignant neoplasm, or death from any cause, whichever occurred first. Overall survival (OS) was defined as time from study enrollment to death from any cause. EFS and OS were censored at the patient’s last contact date and follow-up was current through June 30, 2018. EFS and OS were estimated using the Kaplan–Meier method with confidence intervals (CIs) estimated by the Peto–Peto method [31, 32]. Associations between patient and clinical characteristics and survival were evaluated using log-rank tests, as appropriate. Statistical significance was determined at the 0.05 level with no correction for multiple comparisons. All statistical analyses were conducted using SAS version 9.4.

## 3 | Results

### 3.1 | Patient and Disease Characteristics

Between February 2007 and February 2012, 24 patients with ASPS were enrolled on ARST0332, representing 4.5% of the total 529 eligible patients on the study. Patient and disease characteristics are summarized in Table 1. Patients had a median age of 15.2 years (range 2.8, 23.6 years). The majority of patients had an extremity primary tumor site (71%) and primary tumor diameter > 5 cm (54%). Eleven of 24 patients (46%) had metastatic disease at diagnosis. All patients with metastatic disease had a primary tumor size > 5 cm, had lung metastases, and additional sites of metastases included bone (*n* = 2), liver (*n* = 1), and brain (*n* = 1). No patients had lymph node metastases. ASPS is, by definition, POG grade 3 and all tumors were classified as FNCLCC grade 2 by central pathology review.

### 3.2 | Treatments and Outcomes Across all Patients

**FIGURE 1 |:**
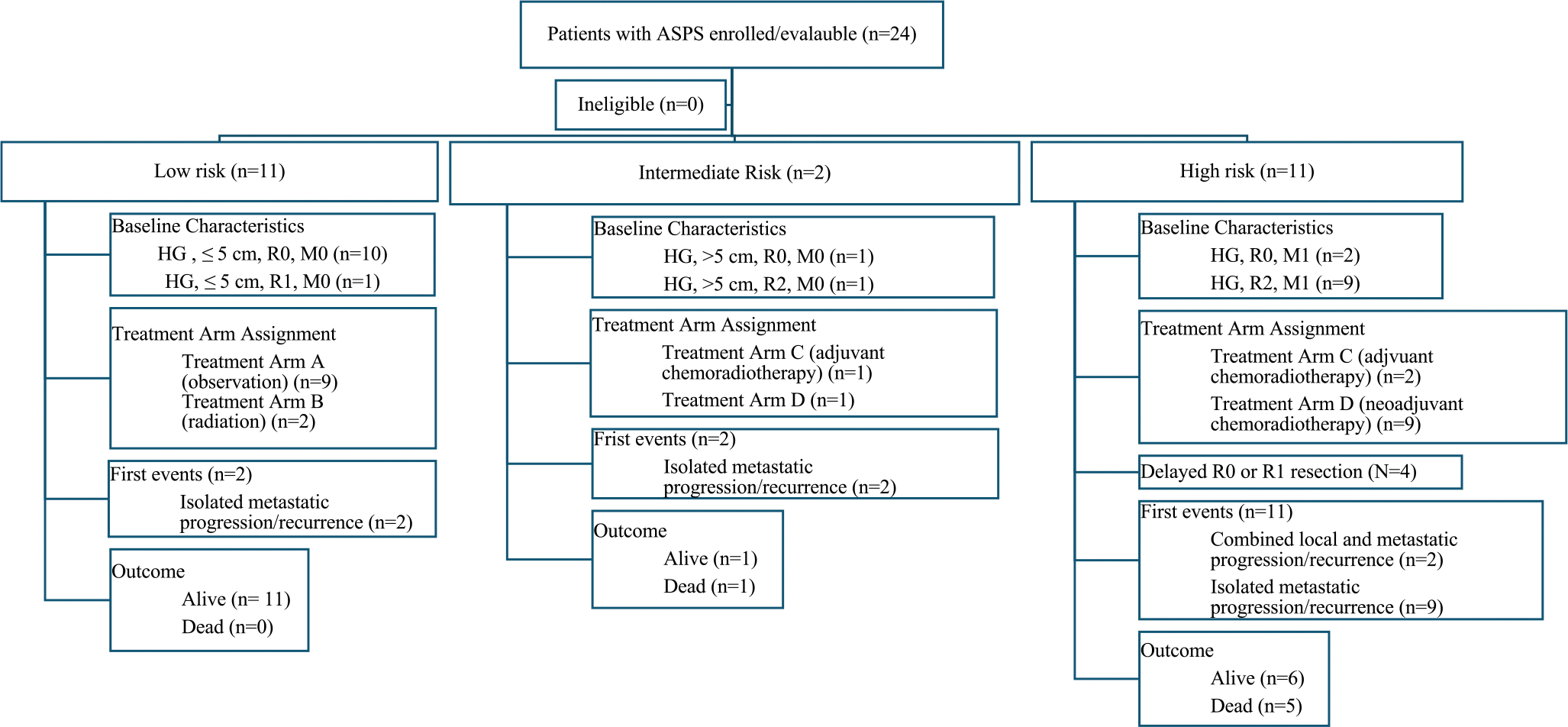
Disease characteristics, treatment, events, and outcomes for patients with ASPS enrolled on ARST0332. Kaplan-Meier curves representing (A) event-free survival by risk group; (B) overall survival for patients in the low-, intermediate-, and high-risk groups; (C) event-free survival by treatment arm; and (D) overall survival by treatment arm.

**FIGURE 2 |:**
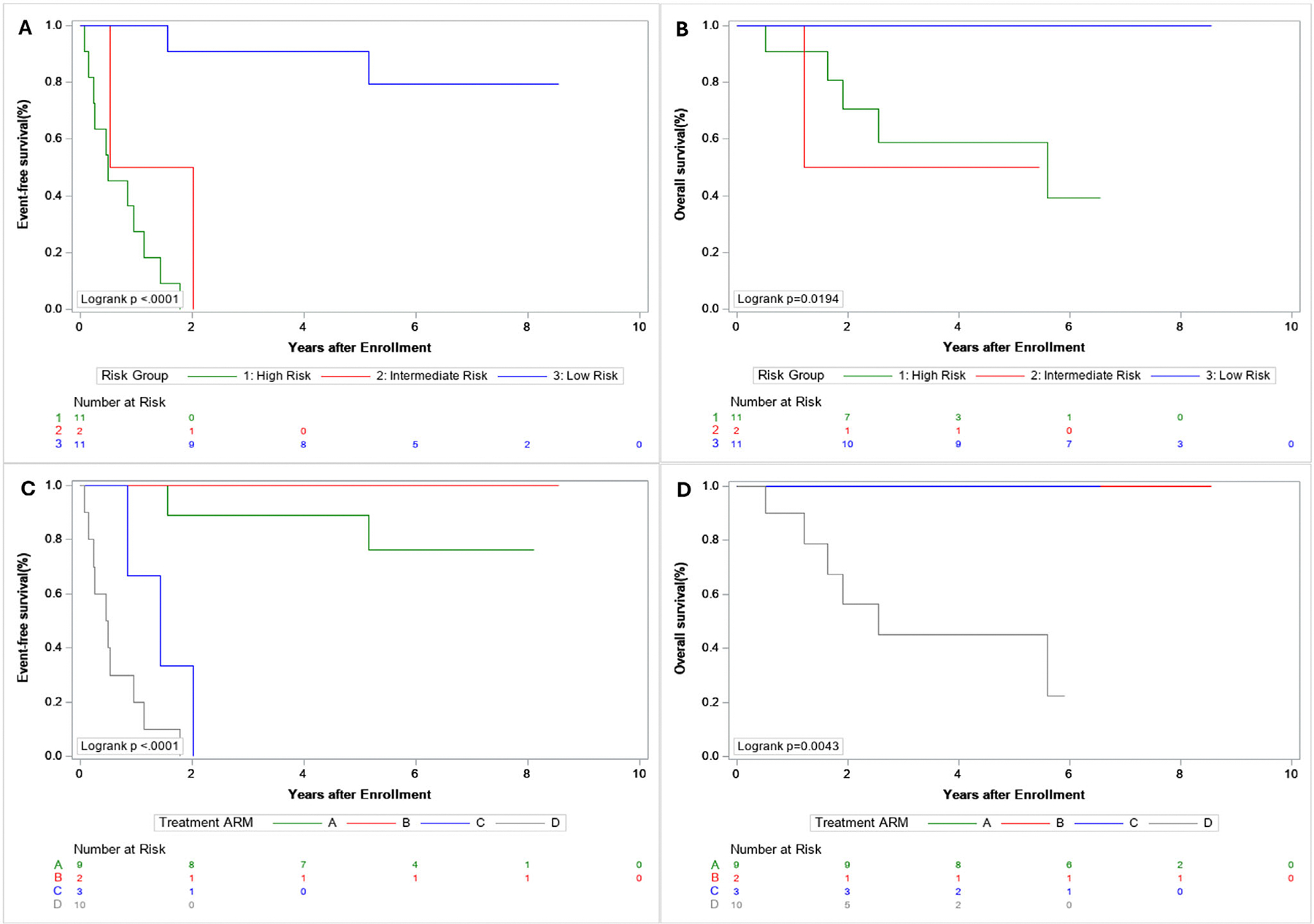
Survival by risk group and treatment arm.

Treatments and outcomes are summarized in Table 2 and Figures 1 and 2. Overall, R0 or R1 resection of the primary tumor was achieved in 19 of 24 patients (79%): 14 of 24 (58%) at study entry and 5 of 24 (21%) after neoadjuvant chemotherapy. Five-year EFS and OS for all patients with ASPS was 41% (95% CI: 19%, 63%) and 78% (95% CI: 58%, 98%), respectively, with median follow-up of 6.9 years (range: 2–8.5 years). As shown in Table 2, 5-year EFS and OS differed significantly by maximal tumor diameter, presence or absence of metastatic disease, risk group, treatment arm, and upfront primary tumor resection status, and timing (upfront vs. delayed) of R0 or R1 resection. Five-year EFS but no OS differed significantly by bone and tumor invasiveness.

Fifteen of 24 patients (62%) experienced a tumor progression or recurrence, all of which were either combined local or distant (*n* = 2) or distant only (*n* = 13), with a median time to progression or recurrence of 0.8 years (range 0.08–5.2 years). Both patients who had a combined local and distant recurrence had received radiation to the primary site. There were no isolated local tumor progressions or recurrence. All patients who experienced an event had the lung as a site of disease progression or recurrence and additional sites included bone (*n* = 2) and thorax (*n* = 1). There were six patient deaths, all of which were due to disease progression, and five of six deaths occurred in patients who presented with metastatic disease at diagnosis.

### 3.3 | Treatments and Outcomes for LR Disease

Eleven of 24 (46%) patients had LR disease. Of these 11 patients, nine were assigned to arm A and treated with surgery only and two were assigned to arm B and treated with surgery and radiation. Patients on arm A had 5-year EFS and OS of 89% and 100%, respectively. Two of the nine patients on arm A had events that were both metastatic relapses, one of which occurred at 6 years. The two patients on arm B had an estimated 5-year EFS and OS of 100%. Across all patients with LR disease, the estimated 5-year EFS and OS was 91% and 100%, respectively.

### 3.4 | Treatments and Outcomes for IR Disease

Two of 24 (8.3%) patients had IR disease. Of the two patients with IR disease, one underwent R0 resection prior to study entry, was assigned to arm C, and completed all protocol therapy with adjuvant chemoradiotherapy. The other patient had a localized tumor > 5 cm that underwent biopsy only prior to study entry, was assigned to arm D, underwent neoadjuvant chemoradiotherapy and had a delayed R0 resection but came off protocol therapy early due to PD. Both patients with IR experienced a first event of metastatic disease progression/relapse. The estimated 5-year EFS and OS for patients with IR disease was 0% and 50%, respectively, although this risk group included only two patients.

### 3.5 | Treatments and Outcomes for HR Disease

Eleven of 24 patients (46%) had HR disease. Two of the 11 patients with HR disease had metastatic disease with R0 resections of the primary site prior at study entry, were assigned to arm C, and both completed protocol therapy with adjuvant chemoradiotherapy. Nine of the 11 patients with HR disease had metastatic disease with unresected primary tumors and were assigned to arm D. Of these nine patients with HR disease on arm D, four of nine (44%) underwent RT followed by resection at week 13, all achieving R0 resections. Of the remaining five patients, two came off study prior to RT and delayed resection, two received RT but came off protocol therapy before delayed resection, and one had definitive RT without surgery. In total, eight of the nine patients with HR disease on arm D came off protocol therapy—four due to PD, three due to physician determination it was in the patient’s best interest, and one due to patient/parent refusal of further therapy. None of the 11 patients with metastatic disease underwent gross total resection of metastases or received RT to metastatic sites. Of these 11 patients, eight came off protocol therapy prior to the end of therapy when local control of metastatic sites was indicated. Of these eight, four came off protocol therapy for reasons other than PD and all later developed isolated PD of existing metastatic sites as their first event and the other four came off protocol therapy early due to PD, one who had isolated PD of existing metastatic site(s) as their first event, one who had PD of existing metastatic site(s) and new metastatic sites as their first event, one who had PD of the primary site, existing metastatic sites, and new metastatic sites, and one who had PD of the primary site and new metastatic sites as their first event. The remaining two patients completed protocol therapy but did not undergo metastatic site local control and both had first disease progression or recurrence in metastatic sites that were present at study entry. The estimated 5-year EFS and OS for patients with HR disease was 0% and 59%, respectively.

### 3.6 | Response to Neoadjuvant Chemoradiotherapy

Tumor response to neoadjuvant chemoradiotherapy was evaluable in six of 10 patients assigned to arm D. Of the six patients evaluable for response to neoadjuvant chemoradiotherapy, three (50%) had SD and three (50%) had PD, thus none had an objective response. The remaining four patients were not evaluable for response due to imaging that was not evaluable (*n* = 1) or discontinuing protocol therapy prior to response assessment for reasons other than PD (*n* = 3).

## 4 | Discussion

Although ASPS accounts for only 1%–2% of all cases of NRSTS in children and young adults, it represented 4.5% of the population enrolled on ARST0332 [33]. The reason(s) for the overrepresentation of ASPS in this study are unclear, but it may be, in part, due to historical lack of clarity on the optimal approach to the treatment of this disease, making a study option particularly appealing. Consistent with prior reports, most cases of ASPS in ARST0332 arose in the extremities (71%) with a high rate (46%) of metastatic disease at diagnosis [1, 9, 11, 12, 26]. All patients with metastatic disease had lung involvement with or without other sites. One patient had brain metastasis. While brain metastases are uncommon in NRSTS, the propensity of ASPS to metastasize to the brain is well documented, particularly in the context of disseminated disease, underscoring the importance of considering brain imaging in this setting [8]. ASPS was considered POG grade 3, regardless of the pathologic features, and FNCLCC grading, which is not generally applied to ASPS, classified all ASPS cases as grade 2, revealing the limitations of both systems in differentiating among ASPS cases. This supports the notion that histologic type is more informative than grade in ASPS and similar entities [34].

Patients with LR ASPS (*n* = 11) had excellent outcomes with a 5-year EFS and OS of 91% and 100%, respectively. Of note, one patient with LR ASPS had a localized tumor ≤ 5 cm with a recorded R0 resection and was treated on arm B, although these features fit arm A. For this analysis, R0 was defined as a complete resection (i.e., no tumor on ink) while the R0 protocol definition required at least 5 mm margins. Thus, it is likely that this patient’s tumor was completely resected with margins < 5 mm (i.e., R0 for analysis but R1 for protocol treatment assignment) with correct assignment to arm B, although data are not available to confirm this. There were a small number (*n* = 2) of patients in the IR group, making it challenging to draw conclusions for that group, although outcomes for IR and HR disease (combined *n* = 13) were suboptimal with all IR and HR patients experiencing disease progression or recurrence despite aggressive multimodal therapy. Notably, all of the progression or recurrences in patients with IR and HR disease were metastatic with or without primary site PD. The median time to progression or recurrence was relatively short (0.8 years). However, the EFS of 0% for the IR and HR is notably different than the 5-year OS for the IR and HR groups of 50% and 59%, respectively. This may be reflective of the indolent nature of ASPS, although we are limited in our ability to draw conclusions due to the lack of details regarding treatments given after progression or relapse or longer follow-up data. Notably, none of the 11 patients with metastatic disease at study entry underwent metastatic site local control at the end of therapy as indicated in the protocol, and five of those patients developed isolated PD or recurrence of metastatic sites present at study entry. Given this and the short median time to progression, it is of interest to evaluate the potential role of early and comprehensive local control of metastatic sites, when feasible.

This analysis confirms that surgical resection remains an essential part of the treatment of ASPS. EFS and OS were significantly higher for patients for whom a primary tumor R0 or R1 resection was achieved compared to those who had R2 or no resection. Given the small number of patients, it is difficult to comment on the role of RT. Neither of the two patients on arm B who received adjuvant RT experienced an event. Of the 13 patients assigned to receive neoadjuvant or adjuvant chemoradiotherapy, 11 received RT and all experienced an event. Of these 11 first events, two were combined local and metastatic progression/recurrences, which both occurred in patients with metastatic disease and who had RT to the primary site. The other nine first events were isolated metastatic progression/recurrences and no one received RT to metastatic sites. This study also confirms poor sensitivity of ASPS to the conventional soft tissue sarcoma chemotherapy combination of ifosfamide and doxorubicin reported from other studies, as none of the six patients evaluable for response to neoadjuvant chemoradiotherapy achieved an objective response. Similarly, on the European Pediatric Soft Tissue Sarcoma Study Group (EpSSG) 2005 study, four of 22 patients with ASPS received chemotherapy, and there were no objective responses [11]. Given the high rate of metastatic disease at diagnosis and distant failures, more effective systemic therapy is needed for ASPS.

Recent literature has shown that immune checkpoint inhibitors and/or tyrosine kinase inhibitors (TKIs) have efficacy in ASPS. Specifically, atezolizumab, a PD-L1 antibody, was approved by the United States Food and Drug Administration for the treatment of adult and pediatric patients with unresectable or metastatic ASPS. This approval was based on the results of a single-arm phase II trial on which 52 adult and pediatric patients with unresectable or metastatic ASPS were treated with atezolizumab, with an objective response of 37% and median progression-free survival (PFS) of 21 months [14]. The anti-PD-1 monoclonal antibody pembrolizumab has also been shown to have activity in ASPS as a single agent and in combination with axitinib [15, 16]. Among 14 patients with ASPS enrolled on a phase 2 trial of pembrolizumab, the objective response rate (ORR) was 57% and an additional 21% of patients had SD [15]. Additionally, on a phase 2 trial of pembrolizumab and axitinib, six of 11 (54%) evaluable patients with ASPS achieved an objective response and two of 11 (18%) achieved SD with a median PFS of 12.4 months [17]. There are additional data on the activity of other immune checkpoint inhibitors, including single agent nivolumab, the combination of nivolumab and ipilimumab, and the combination of durvalumab and tremelimumab [18–21]. The TKIs pazopanib [22, 23], cabozantinib [24], sunitinib [25, 35, 36], and cediranib [25, 37–41] have also all shown clinical activity in ASPS. On the basis of these data, the current preferred systemic therapy approaches for unresectable and/or metastatic ASPS include immune checkpoint inhibitors and/or TKIs, although there are no head-to-head data to guide prioritization of agent(s) within these classes.

In summary, ASPS has a high rate of metastatic disease at diagnosis, is resistant to ifosfamide and doxorubicin, and metastatic progression or recurrence accounts for the majority of treatment failures. The data from ARST0332, including the excellent outcomes with surgery with or without RT in patients with LR ASPS and the lack of objective response to chemoradiotherapy in IR and HR ASPS, along with prior studies including EpSSG 2005 and activity of immune checkpoint inhibitors (ICIs) and TKIs solidify that ifosfamide and doxorubicin do not have a role in the treatment of ASPS. Further improvements in outcomes are needed for patients with metastatic ASPS.

## Supplementary Material

supplemental file **Supporting Figure 1:** ARST0332 risk group and treatment assignment schema for patients with ASPS.
**Supplemental Table 1:** ARST0332 treatment details for patients with ASPS

Additional supporting information can be found online in the Supporting Information section.

## Figures and Tables

**TABLE 1 |:** Patient and baseline disease characteristics of patients with ASPS enrolled on ARST0332.

Characteristic	Total (*N* = 24)

Age, median (range), years	15.2 (2.8, 23.6)
Sex, *n* (%)	
Female	12 (50%)
Male	12 (50%)
Race, *n* (%)	
White	14 (58%)
Black or African American	7 (29%)
Other/unknown	3 (13%)
Ethnicity, *n* (%)	
Hispanic	5 (21%)
Non-Hispanic/unknown	19 (79%)
Primary tumor site, *n* (%)	
Body wall	4 (17%)
Extremity	17 (71%)
Head and neck	2 (8%)
Visceral	1 (4%)
Maximal tumor diameter, median (range), cm	4.9 (0.5, 22.0)
Maximal tumor diameter, *n* (%)	
≤ 5 *χμ*	11 (46%)
> 5 cm	13 (54%)
Primary tumor depth, *n* (%)	
Deep	20 (83%)
Superficial	4 (17%)
POG grade, *n* (%)	
Grade 3 (high)	24 (100%)
FNCLCC grade, *n* (%)	
Grade 2 (high)	24 (100%)
Bone invasiveness, *n* (%)	
Non-invasive	11 (46%)
Touching	3 (12.5%)
Destroying	3 (12.5%)
Missing	7 (29%)
Neurovascular invasiveness, *n* (%)	
Non-invasive	11 (46%)
Touching	2 (8%)
Surrounding	3 (13%)
Indeterminate	1 (4%)
Missing	7 (29%)
Metastases, *n* (%)	
No	13 (54%)
Yes	11 (46%)
Sites of metastases, *n* (%)	
Bone	2 (8%)
Lung	11 (46%)
Liver	1 (4%)
Brain	1 (4%)

**TABLE 2 |:** Univariate analysis of survival of patients with ASPS enrolled on ARST0332 by clinical features, treatment, and response to treatment.

Stratum	No. at risk	5-year EFS (95% CI)	*p*-value	5-year OS (95% CI)	*p*-value	% with metastatic failure (*N* = 15)	Fisher exact *p*-value

Age							
1–9 years	4	75% (32%, 100%)	0.1	100% (100%, 100%)	0.4	1 (6.7%)	0.1
10–17 years	12	50% (19%, 80%)		73% (43%, 100%)		7 (47%)	
≥ 18 years	8	. (.,.)		73% (30%, 100%)		7 (47%)	
Sex							
Female	12	41% (14%, 69%)	0.8	81% (54%, 100%)	0.4	7 (47%)	>0.9
Male	12	40% (4.9%, 75%)		75% (45%, 100%)		8 (53%)	
Maximal tumor diameter							
≤ 5 cm	11	91% (72%, 100%)	<0.0001	100% (100%, 100%)	0.005	2 (13%)	<0.0001
> 5 cm	13	0.0% (.,.)		57% (21%, 94%)		13 (87%)	
Bone invasiveness							
Non-invasive	11	64% (30%, 97%)	0.01	91% (69%, 100%)	0.4	4 (44%)	0.2
Touching	3	33% (0.0%, 87%)		67% (13%, 100%)		2 (22%)	
Destroying	3	0.0% (.,.)		67% (0.0%, 100%)		3 (33%)	
Neurovascular invasiveness							
Non-invasive	11	54% (22%, 87%)	0.001	82% (54%, 100%)	0.8	5 (56%)	>0.99
Touching	2	. (.,.)		100% (100%, 100%)		1 (11%)	
Surrounding	3	33% (0.0%, 87%)		67% (13%, 100%)		2 (22%)	
Indeterminate	1	0.0% (.,.)		.(.,.)		1 (11%)	
Metastases							
No	13	76% (50%, 100%)	<0.0001	92.% (76%, 100%)	0.02	4 (27%)	0.0006
Yes	11	0.0% (.,.)		59% (16%, 100%)		11 (73%)	
Risk group							
Low risk	11	91% (72%, 100%)	<0.0001	100% (100%, 100%)	0.01	2 (13%)	<0.0001
Intermediate risk	2	0.0% (.,.)		50% (0.0%, 100%)		2 (13%)	
High risk	11	0.0% (.,.)		59% (16%, 100%)		11 (73%)	
Treatment arm							
A	9	89% (67%, 100%)	<0.0001	100% (100%, 100%)	0.004	2 (13%)	<0.0001
B	2	100% (100%, 100%)		100% (100%, 100%)		0	
C	3	0.0% (.,.)		100% (100%, 100%)		3 (20%)	
D	10	0.0% (.,.)		45% (0.0%, 91%)		10 (67%)	
Extent of surgical resection of primary site (upfront)							
R0	13	68% (40%, 97%)	<0.0001	100% (100%, 100%)	0.001	5 (33%)	0.002
R1	1	100% (100%, 100%)		100% (100%, 100%)		0	
R2	10	0.0% (.,.)		45% (0.0%, 91%)		10 (67%)	
Extent of surgical resection of primary site (upfront or delayed)							
R0	18	49% (23%, 75%)	0.0002	87% (69%, 100%)	0.06	10 (67%)	0.08
R1	1	100% (100%, 100%)		100% (100%, 100%)		0	
R2	5	0.00% (.,.)		40% (0.0%, 100%)		5 (33%)	
Timing of R0 or R1 resection of primary site (excluding missing/R2)							
Upfront	14	7% (44%, 97%)	<0.0001	100% (100%, 100%)	0.0002	5 (50.0%)	0.03
Delayed	5	0.0% (.,.)		50% (0.0%, 100%)		5 (50.0%)	
Treatment response at week 13 (patients in arm d only)							
PD	3	0.0% (.,.)	0.1	.(.,.)	0.2	3 (33.3%)	
SD	3	0.0% (.,.)		100% (100%, 100%)		3 (33.3%)	

## Abbreviations

ASPS: alveolar soft part sarcoma
CI: confidence intervals
COG: Children’s Oncology Group
EFS: event-free survival
EpSSG: European Pediatric Soft Tissue Sarcoma Study Group
HR: high risk
ICI: immune checkpoint inhibitor
IR: intermediate risk
LR: low risk
ORR: objective response rate
OS: overall survival
PD: progressive disease
PFS: progressive-free survival
POG: Pediatric Oncology Group
PR: partial response
RT: radiotherapy
SD: stable disease
TKI: tyrosine kinase inhibitor
