# Supplementary material for: Alveolar Soft Part Sarcoma in Pediatric and Young Adult Patients: A Report From the Children’s Oncology Group Study ARST0332
Source: Pediatr Blood Cancer. Author manuscript; Available in PMC 2026 May 13. (PMC13170099; doi:10.1002/1545-5017.70228)
Supplement: supplemental file — Supporting Figure 1: ARST0332 risk group and treatment assignment schema for patients with ASPS. Supplemental Table 1: ARST0332 treatment details for patients with ASPS [file NIHMS2167969-supplement-supplemental_file.docx]

**Supplemental Figure S1: ARST0332 risk group and treatment assignment schema for patients with ASPS**

**
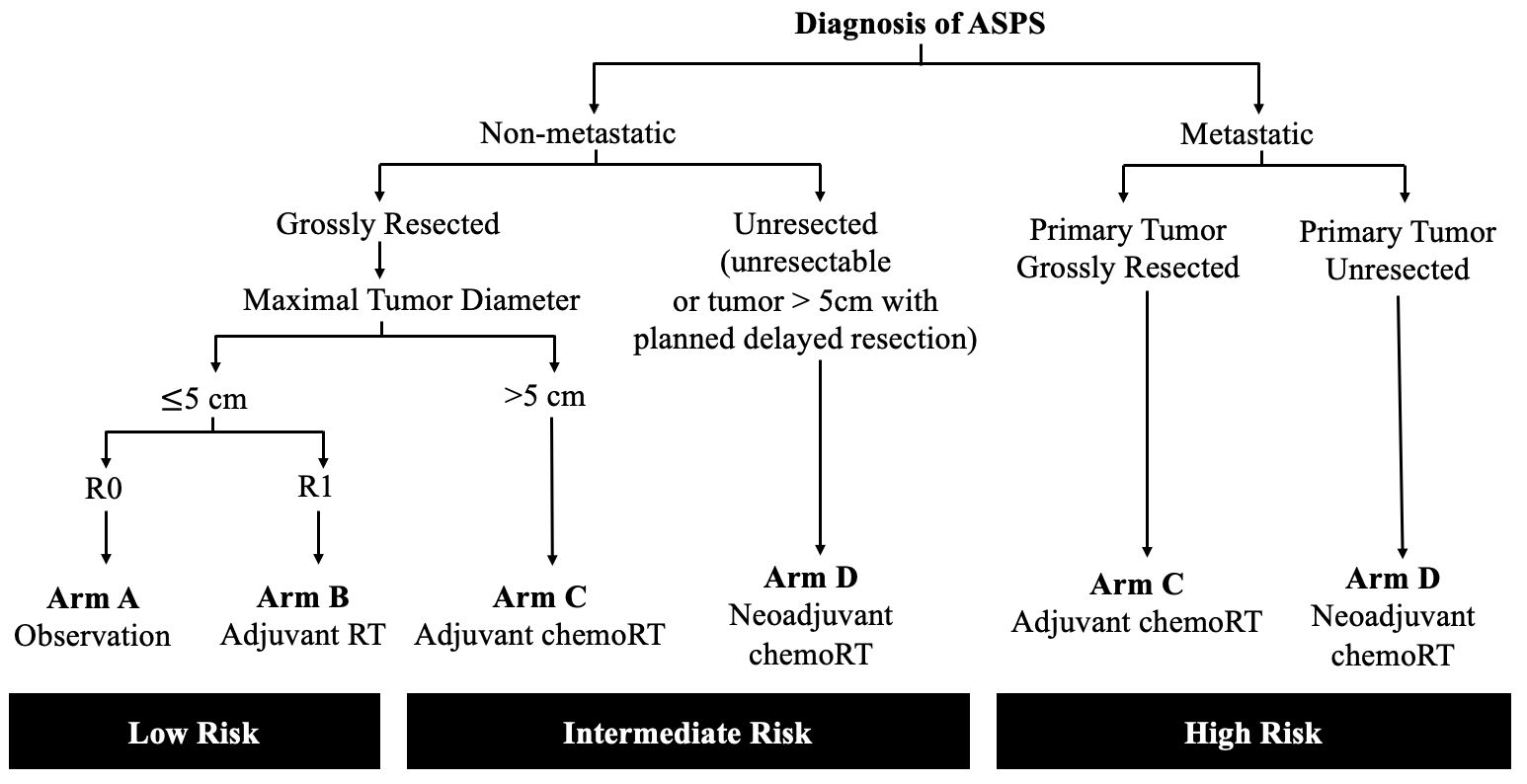
**

Treatment Arm and Risk Group Assignments. The general ARST0332 schema has been simplified to reflect the assignment pathways for ASPS which is classified as high grade on the POG grading system. R0 = complete resection; R1 = microscopic residual disease; RT = radiotherapy.

**Supplemental Table S1: ARST0332 treatment details for patients with ASPS**

| Primary site surgery prior to study entry with gross total resection | **Treatment Arm A: Observation** | | | | | | | | |
| --- | --- | --- | --- | --- | --- | --- | --- | --- | --- |
|  | **Treatment Arm B: Adjuvant radiotherapy**^1^ | | | | | | | | |
|  | **Treatment Arm C: Adjuvant chemoradiotherapy** | | | | | | | | |
|  | Week 1 | Week 4 | Week 7 | Week 10 | Week 13 | Week 16 | Week 19 |  | |
|  | Ifosfamide  Doxorubicin | Ifosfamide  Doxorubicin | Ifosfamide | Ifosfamide | Ifosfamide  Doxorubicin^2^ | Ifosfamide  Doxorubicin^2^ | Doxorubicin |  | |
|  |  | Radiotherapy^3^ | |  | | | | Surgery and/or radiotherapy to metastases^3^ | |
| Primary tumor not grossly resected prior to study entry | **Treatment Arm D: Neoadjuvant chemoradiotherapy** | | | | | | | | |
|  | Week 1 | Week 4 | Week 7 | Week 10 | Week 13 | Week 16 | Week 19 | Week 22 |  |
|  | Ifosfamide  Doxorubicin | Ifosfamide  Doxorubicin | Ifosfamide | Ifosfamide |  | Ifosfamide  Doxorubicin | Ifosfamide  Doxorubicin^4^ | Doxorubicin |  |
|  |  | Radiotherapy^5^ | | | Surgery | Radiotherapy^6^ | |  | Surgery and/or radiotherapy to metastases^4^ |

Treatment details for ARST0332. Ifosfamide: 3 g/m2/dose (1.5 g/m2/dose for age <1 year) IV on days 1, 2, and 3; given with mesna and Doxorubicin: 37.5 mg/m2/dose (18.75 g/m2/dose for age <1 year) IV on days 1 and 2 (maximum 75 mg/dose). Myeloid growth factor was required with chemotherapy except when doxorubicin was administered alone. ^1^ radiotherapy dose for Arms B and C: 55.8 Gy in 31 fractions; ^2^Week 16 and 19 doxorubicin may be moved to weeks 7 and 10 in patients not receiving radiotherapy; ^3^Surgery and/or radiotherapy for metastatic disease present at study entry was recommended at the end of therapy. Gross total resection was recommended when feasible and radiotherapy was recommended for gross or microscopic residual metastatic disease; ^4^Week 19 doxorubicin moved to week 25 in patients receiving radiotherapy after week 13 surgery; ^5^Arm D pre-operative radiotherapy dose: 45 Gy in 25 fractions; ^6^Arm D post-operative radiotherapy for microscopic (10.8 Gy in 6 fractions) or gross residual tumor (19.8 Gy in 11 fractions) administered at the discretion of the radiation oncologist.
